# Genetic Study of White Matter Integrity in UK Biobank (N=8448) and the Overlap With Stroke, Depression, and Dementia

**DOI:** 10.1161/STROKEAHA.118.020811

**Published:** 2018-05-11

**Authors:** Loes C.A. Rutten-Jacobs, Daniel J. Tozer, Marco Duering, Rainer Malik, Martin Dichgans, Hugh S. Markus, Matthew Traylor

**Affiliations:** 1 From the Department of Clinical Neurosciences, Stroke Research Group, University of Cambridge, United Kingdom (L.C.A.R.-J., D.J.T., H.S.M., M.T.); 2 Institute for Stroke and Dementia Research, Klinikum der Universität München, Germany (M. Duering, R.M., M. Dichgans); 3 German Center for Neurodegenerative Diseases, Population Health Sciences, Bonn, Germany (L.C.A.R.-J.).

**Keywords:** cerebral small vessel diseases, diffusion tensor imaging, genetic association studies, humans, white matter

## Abstract

**Background and Purpose—:**

Structural integrity of the white matter is a marker of cerebral small vessel disease, which is the major cause of vascular dementia and a quarter of all strokes. Genetic studies provide a way to obtain novel insights in the disease mechanism underlying cerebral small vessel disease. The aim was to identify common variants associated with microstructural integrity of the white matter and to elucidate the relationships of white matter structural integrity with stroke, major depressive disorder, and Alzheimer disease.

**Methods—:**

This genome-wide association analysis included 8448 individuals from UK Biobank—a population-based cohort study that recruited individuals from across the United Kingdom between 2006 and 2010, aged 40 to 69 years. Microstructural integrity was measured as fractional anisotropy- (FA) and mean diffusivity (MD)-derived parameters on diffusion tensor images. White matter hyperintensity volumes (WMHV) were assessed on T2-weighted fluid-attenuated inversion recovery images.

**Results—:**

We identified 1 novel locus at genome-wide significance (*VCAN* [versican]: rs13164785; *P*=3.7×10^−18^ for MD and rs67827860; *P*=1.3×10^−14^ for FA). LD score regression showed a significant genome-wide correlation between FA, MD, and WMHV (FA-WMHV rG 0.39 [SE, 0.15]; MD-WMHV rG 0.56 [SE, 0.19]). In polygenic risk score analysis, FA, MD, and WMHV were significantly associated with lacunar stroke, MD with major depressive disorder, and WMHV with Alzheimer disease.

**Conclusions—:**

Genetic variants within the *VCAN* gene may play a role in the mechanisms underlying microstructural integrity of the white matter in the brain measured as FA and MD. Mechanisms underlying white matter alterations are shared with cerebrovascular disease, and inherited differences in white matter microstructure impact on Alzheimer disease and major depressive disorder.

## Abstract 2

Supplemental Digital Content is available in the text.

Cerebral small vessel disease (cSVD) is the major cause of vascular dementia and the pathology underlying a quarter of all strokes in the form of small subcortical (lacunar) strokes or intracerebral hemorrhage (ICH). Despite these numbers, the pathogenesis of cSVD is largely unknown, and this knowledge gap is a major factor behind the lack of specific therapies to delay cSVD progression.^1,2^

The most commonly studied marker of cSVD is white matter hyperintensities (WMHs) on magnetic resonance imaging (MRI). The prevalence and severity of WMH increases with advancing age and is associated with cardiovascular risk factors, stroke, dementia, and depression.^3,4^ However, studies suggest that microstructural tissue alterations underlying the cerebral white matter (fractional anisotropy [FA] and mean diffusivity [MD]) detected using diffusion tensor imaging (DTI) are more predictive of cognitive decline.^5^

Small vessels in the brain are difficult to investigate in vivo. Genetic studies provide a way to obtain novel insights in the disease mechanism underlying cSVD. Previous genome-wide association studies (GWAS) in population-based individuals and patients with stroke identified 12 genome-wide significant loci associated with WMH.^6,7^ However, there are only few published GWAS with a limited sample size that investigate the microstructural integrity of the white matter.^8–10^ The aims of this analysis were 2-fold: (1) to identify genetic variants associated with microstructural integrity of the white matter (FA and MD) in 8448 population-based individuals in UK Biobank and (2) to elucidate the relationships of FA, MD, and white matter hyperintensity volumes (WMHV) with clinical end points (eg, stroke, major depressive disorder, and Alzheimer disease).

## Methods

The genetic and phenotypic UK Biobank data are available on application to the UK Biobank (http://www.ukbiobank.ac.uk/).

### Study Population and Ethical Approval

UK Biobank is a prospective study that recruited 502 620 community-dwelling participants from across the United Kingdom between 2006 and 2010, aged 40 to 69 years (http://www.ukbiobank.ac.uk). The study collects extensive data from questionnaires, interviews, health records, physical measures, biological samples, and imaging.

A subset of the participants also underwent brain MRI. In the present study, we used the second release of MRI data, which included 9066 subjects who underwent brain MRI, on average 6.6 years (SD, 1.0 years) after initial recruitment at mean age 55.5 years (SD, 7.4 years) and had usable T2-weighted fluid-attenuated inversion recovery or DTI images. Patients with a baseline diagnosis of stroke, multiple sclerosis, Parkinson disease, dementia, any other neurodegenerative problem (*InternationalClassification of Diseases, Ninth Revision/Tenth Revision*, or self-report or health-record linkage) or no genetic data were excluded (Table I in the online-only Data Supplement). Participants with consistently extreme outlying tract-averaged water diffusion biomarker values were removed after visual inspection of the data by the authors.

UK Biobank received ethical approval from the research ethics committee (reference 11/NW/0382). All participants provided informed consent to participate. The present analyses were conducted under UK Biobank application number 19463.

### Magnetic Resonance Imaging

Procedures for brain imaging acquisition and initial quality check have been described previously and are available on the UK Biobank website (Brain Imaging Documentation V1.3; http://www.ukbiobank.ac.uk).^11^

In brief, all brain MRI data were acquired on a single standard Siemens Skyra 3T scanner (Siemens Medical Solutions, Germany) using the standard Siemens 32-channel radiofrequency receiver head coil. Sagittal T1-weighted scans were acquired using a 3-dimensional magnetization-prepared rapid acquisition gradient-echo sequence (resolution, 1×1×1 mm; field of view, 208×256×256; inversion time/repetition time=880/2000 ms). Sagittal T2-weighted fluid-attenuated inversion recovery scans were obtained using a 3-dimensional SPACE sequence (resolution, 1.05×1.0×1.0 mm; field of view, 192×256×256; inversion time/repetition time=1800/5000 ms). DTI scans were acquired with a spin-echo echo-planar imaging sequence and multishell acquisition (b0=0 s/mm^−2^, b=1000 s/mm^−2^, and b=2000 s/mm^−2^; 100 distinct diffusion-encoding directions [50 in each shell]; 2-mm isotropic voxels; field of view, 104×104×72).

### White Matter Hyperintensities

WMHs were automatically segmented using the combined T1 and T2-weighted fluid-attenuated inversion recovery data as input in the Brain Intensity Abnormality Classification Algorithm tool.^12^ Brain Intensity Abnormality Classification Algorithm is a fully automated supervised method for WMH detection, based on the k-nearest neighbor algorithm, which gives the probability per voxel of being WMH. The total WMHV was calculated from the voxels exceeding a probability of 0.9 of being WMH and located within a white matter mask. Obtained values were adjusted for the total intracranial volume and log transformed because of their skewed distribution.

### FA and MD

After gradient distortion correction and further correction for head movement and eddy currents, diffusion tensors and scalar diffusion parameters (ie, FA and MD) were calculated using the b=1000 shell (50 directions) and DTIFIT from the FSL software.^13^ The FA maps produced were then fed into tract-based spatial statistics processing, which aligns the FA map onto a standard-space FA template; in this work, the standard FMRIMB_158_FA template was used as the target image. The standard space FA subject images were then skeletonized and the MD (and other DTI output) maps were projected onto the subject skeleton, using the FA-derived alignment parameters. A set of 48 standard space tracts have been defined previously,^14^ these are then used as masks to generate tract-specific masks from the skeletonized images. These are then used to produce a mean FA or MD from each tract for each subject. This is similar to the processing applied in the ENIGMA project (http://enigma.ini.usc.edu/protocols/dti-protocols).^14,15^

Principal component analysis was applied on the 48 tracts to extract a latent measure. The first principal component of FA (FA.PC1) and MD (MD.PC1) was used in subsequent analyses as dependent variable.

### Genetic Data

We used the June 2017 release of the imputed genetic data from UK Biobank (downloaded on June 3, 2017). Details of the design of the arrays, sample processing, and stringent quality control have been described elsewhere.^16^ In brief, 2 closely related arrays from Affymetrix, the UK BiLEVE Axiom array (9.9% of individuals), and the UK Biobank Axiom array were used to genotype ≈805 426 markers with good genome-wide coverage. Phasing was performed using SHAPEIT3 and imputation to a merged Haplotype Reference Consortium reference panel (39 131 578 autosomal single-nucleotide polymorphisms [SNPs]) and UK10K and 1000 Genomes Phase 3 panel was performed using the IMPUTE4 package.^16–18^ Imputed genotypes were available for 487 442 individuals in this study.^16^ From the resulting data set, we excluded (1) individuals who did not segregate with European samples based on principal component analysis, (2) individuals with high-level heterozygosity and missingness (>5%), and (3) individuals whose reported sex was inconsistent with sex inferred from the genetic data. In addition, only SNPs imputed from the HRC panel were included in this analysis.

### Statistical Analysis

In this analysis, we first subset the genetic data on the individuals who also had MRI imaging data. We performed a GWAS of FA, MD, and log (WMHV), using SNPTEST v2.5.4-beta3, including age at MRI, sex, genotyping batch, and the first 10 ancestry informative principal components as covariates. We set the study-wide significance threshold at *P*<1.7e-8, accounting for the 3 phenotypes studied. At this threshold, we had 80% power to identify variants explaining >0.5% of the trait variance.

### Fine-Mapping Derived From Credible SNP Set Analyses

For all SNPs in linkage disequilibrium (LD) with the lead SNP (r2>0.1), we calculated Bayes factors from the effect sizes and SEs using Wakefield approximation.^19^ We then used these Bayes factors to calculate the posterior probability that each variant is causal and the 95% credible set for each association (the smallest set of variants with posteriors that sum to at least 95%) as described in the study by Maller et al.^20^

To identify additional independent signals at genome-wide significant loci we performed a forward stepwise regression using SNPTEST.

### Functional Annotation

To evaluate whether the genome-wide significant variants potentially influence gene expression, we examined genome-wide cis-expression quantitative trait loci (eQTL) data in multiple tissues from 3 major eQTL databases: the Blood eQTL Browser,^21^ the Genotype-Tissue Expression Project,^22^ and the Brain eQTL Almanac.^23^

We performed a lookup of the genome-wide significant SNPs in available GWAS summary statistics of relevant clinical end points (Alzheimer disease,^24^ major depressive disorder,^25^ ICH,^26^ and MRI-confirmed lacunar stroke^27^), of which details are provided in Table II in the online-only Data Supplement and Methods in the online-only Data Supplement.

### Heritability and Genetic Correlations With Related Traits

We estimated the heritability of FA, MD, and WMHV and tested for genetic correlation between the white matter measures using LD score regression.^28^ Subsequently, we estimated the genetic correlation between the white matter measures and 4 clinical end points: Alzheimer disease, major depressive disorder, ICH-, and MRI-confirmed lacunar stroke. Concerning stroke subtypes, specifically ICH- and MRI-confirmed lacunar stroke, were selected because small vessel disease is presumed to be the most important in these stroke subtypes. These analyses were based on genome-wide summary statistics obtained from online repositories and locally available data (Table III in the online-only Data Supplement).

### Polygenic Association of FA, MD, and WMHV With Clinical End Points

We derived polygenic risk scores at 3 different levels of significance (*P*<0.0001, *P*<0.05, *P*<0.5) from the GWAS data of FA, MD, and WMHV and tested them for association with Alzheimer disease, major depressive disorder, ICH-, and MRI-confirmed lacunar stroke using an inverse-variance weighted method using summary statistics data. We derived an independent (r^2^ <0.1 or 500 Kb apart) set of SNPs at each threshold using an LD-clumping procedure used using plink v1.90b3.45. Risk score analysis was performed in R using the gtx package.

## Results

In total, 8448 individuals were included in the present analysis (Figure I in the online-only Data Supplement).

At the time of the MRI scan, mean age was 62.2 (7.4) years. Among the individuals who passed genetic quality control checks, FA and MD measures could be calculated in 8239 individuals, and WMHV were available in 8429. The 3 measures were heritable (*h*^*2*^=0.29 for FA, *h*^*2*^=0.17 for MD, and *h*^*2*^=0.18 for WMHV) and showed high phenotypic and genetic correlation (Table 1).

**Table 1.**
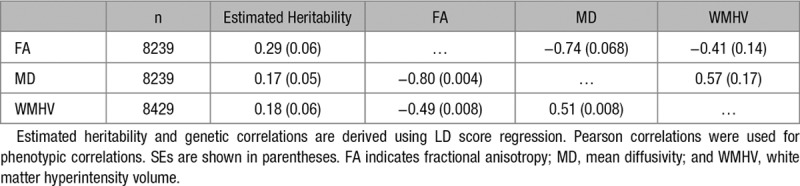
Estimated Heritability, Phenotypic (Below Diagonal), and Genetic (Above Diagonal) Correlations Between the UK Biobank Magnetic Resonance Imaging Variables

### Genome-Wide Association Analysis of FA and MD

**Figure 1.**
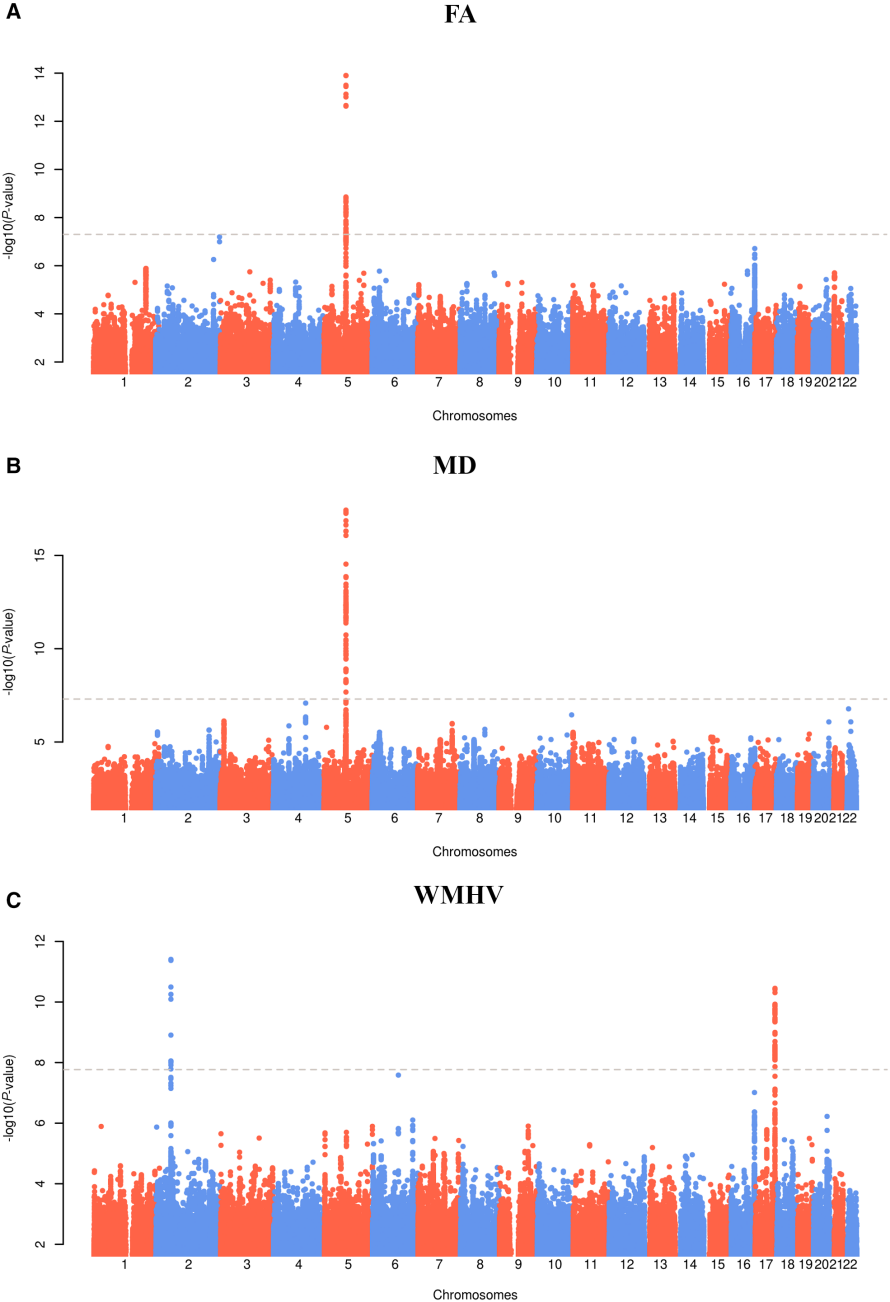
Association of genome-wide single-nucleotide polymorphisms with fractional anisotropy (FA; **A**) and mean diffusivity (MD; **B**) by genomic position. Association results for the genome-wide association analysis for FA (**A**), MD (**B**), and white matter hyperintensity volumes (WMHV; **C**). The dashed line marks the threshold of statistical significance (*P*=1.7×10^−8^).

Table 2 shows the genome-wide significant loci (*P*<1.7×10^−8^) for FA, MD, and WMHV with their corresponding alleles and effect sizes. The inflation of test statistics (λ) was equal to the inflation expected for the sample size. Manhattan plots and Q-Q plots are displayed in Figure 1 and Figure II in the online-only Data Supplement, respectively. Regional plots for the genome-wide significant hits for FA, MD, and WMHV are provided in Figures III, IV, and V in the online-only Data Supplement.

**Table 2.**
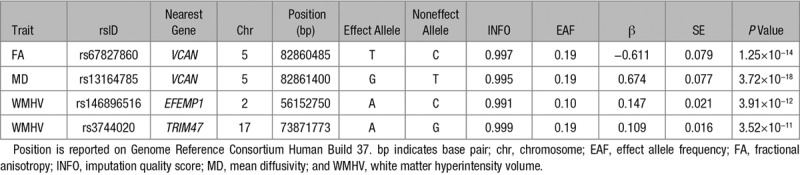
Genome-Wide Significant Loci Associated With FA, MD, and WMHV

We identified 1 region harboring genome-wide significant SNPs for FA or MD. This locus, which was shared between FA and MD, was on chr5q14 (top SNP for FA: rs67827860, *P*=1.3×10^−14^; top SNP for MD: rs13164785, *P*=3.7×10^−18^; LD r^2^=1 for these 2 SNPs).

We constructed 95% credible sets for the chr5q14 locus, which return the set of SNPs in which the causal SNP is contained with 95% certainty. The 95% credible set for the chr5q14 association contained 6 SNPs (rs13164785, rs67827860, rs10052710, rs17205972, rs12653308, and rs3852188).

To identify additional independent signals at the genome-wide significant loci, we performed a forward stepwise regression using SNPTEST on both regions, which yielded a second independent genome-wide signal for both FA and MD in the chr5q14 region (Figures VI and VII in the online-only Data Supplement). Results of the joint model containing both SNPs are given in Table IV in the online-only Data Supplement.

The chr5q14 locus contained 33 genome-wide SNPs for FA and 116 genome-wide SNPs for MD, of which 31 overlapped. The top SNP at the chr5q14 locus maps to an intron of the gene *VCAN*.

Functional annotation of the genome-wide significant SNPs in the chr5q14 region using the Genotype-Tissue Expression and Brain eQTL Almanac resources identified no significant eQTLs at genome-wide significance. However, there was significant cis association with expression of *VCAN* in blood (rs3852188; *P*=1.65×10^−5^).

A nominally significant SNP for the chr5q14 locus (rs3852188; *P*=0.01) was found in the MRI-confirmed lacunar stroke data set, but the effect was in the opposite direction. No other significant associations for any of the traits were found.

Two genome-wide significant loci were identified for WMHV located at 2p16 (top SNP rs146896516; *P*=3.91×10^−12^) and 17q25 (top SNP rs3744020; *P*=3.52×10^−11^; Table 2). Both loci are known WMH loci, reported previously in population-based and stroke population.

Both SNPs showed the expected direction of effect in the association with both FA and MD, but only rs146896516 was nominally associated with MD (Table V in the online-only Data Supplement). The top SNPs for MD and FA at the chr5q14 locus were nominally associated with WMHV (*P*=3.51×10^−6^ and *P*=2.95×10^−6^, respectively).

### Genetic Correlation With Related Traits Using LD Score Regression

**Figure 2.**
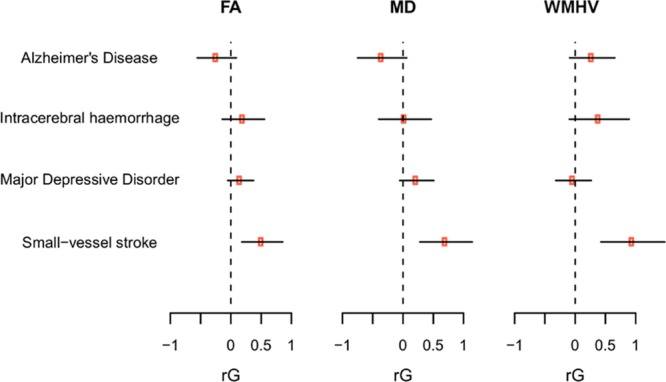
LD score regression results of white matter measures and related clinical phenotypes. Shared genetic contribution between the white matter phenotypes and related clinical phenotypes as determined LD score regression analysis. Genetic correlation (rG) and 95% confidence intervals are shown. The correlation estimates for fractional anisotropy (FA) have been inverted to show trait raising risk (ie, a positive rG means that the other trait is positively associated with a reduction in FA). MD indicates mean diffusivity; and WMHV, white matter hyperintensity volume.

Genetic correlation between the MRI phenotypes and related phenotypes was estimated using LD score regression. The Bonferroni-adjusted significance threshold for this analysis was set at *P*=0.004. There was significant positive genetic correlation between MRI-confirmed lacunar stroke and MD (rG [SE]=0.71 [0.22]; *P*=0.0013), FA (rG [SE]=0.52 [0.17]; *P*=0.0028), as well as WMHV (rG [SE]=0.90 [0.27]; *P*=0.0004). All other correlations were not significant (Figure 2).

### Polygenic Association of FA and MD With Clinical End Points

**Figure 3.**
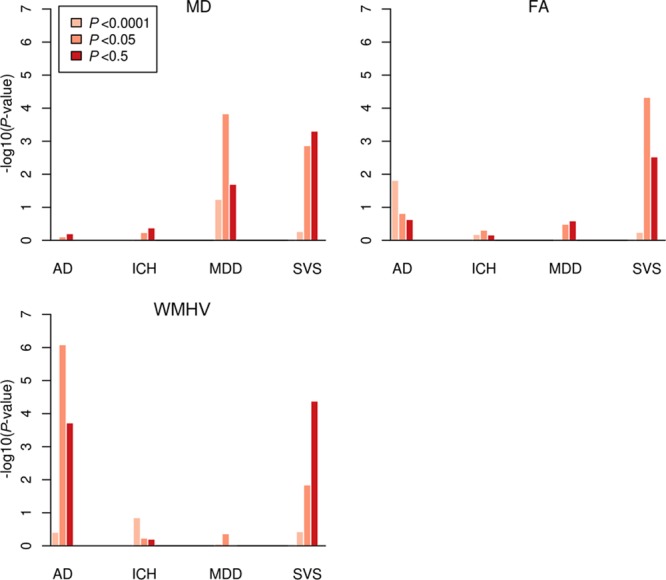
Association between polygenic risk scores of white matter measures and clinical end points. AD indicates Alzheimer disease; FA, fractional anisotropy; ICH, intracerebral hemorrhage; MD, mean diffusivity; MDD, major depressive disorder; SVS, magnetic resonance imaging–confirmed lacunar stroke; and WMHV, white matter hyperintensity volume.

We further tested FA, MD, and WMHV polygenic risk scores (*P*<0.0001, *P*<0.05, and *P*<0.5) for association with clinical end points. There was a polygenic association of all 3 MRI traits with MRI-confirmed lacunar stroke, with a similar association across all traits (Figure 3). In addition, MD polygenic risk scores were significantly associated with major depressive disorder. WMHV polygenic risk scores showed a significant association with Alzheimer disease.

## Discussion

We performed a GWAS of brain white matter microstructural integrity as assessed on DTI, identifying a novel genome-wide significant locus on chr5q14. We found strong evidence for a shared genetic component between FA, MD, and WMHV. Furthermore, we demonstrated that genetic variants that influence all white matter measures studied confer risk of lacunar stroke, as well as demonstrating associations between MD—a marker of white matter ultrastructural damage and major depressive disorder—and between Alzheimer disease and WMHV.

The top SNP within the chr5q14 locus maps to an intron of *VCAN*, which encodes the extracellular matrix proteoglycan VCAN. The top SNPs were only significantly associated with expression of *VCAN* in blood. VCAN is a versatile protein, which plays a role in intercellular signaling and in connecting cells with the extracellular matrix.^29^ Furthermore, VCAN may play a role in the regulation of cell motility, growth, and differentiation.

SNPs in chr5q14 have been linked to intracranial aneurysm of which one of the lead SNPs in the previous study is among our genome-wide significant SNPs (rs173686; *P*=3.32×10^−10^).^30^

Furthermore, mutations in chr5q14 have been linked to Wagner disease—a rare vitreoretinal degeneration inherited as an autosomal dominant trait. VCAN is a component of the vitreous and likely involved in the maintenance and structural integrity of the vitreous.^31^ Mutations linked to Wagner disease lead to alternative splicing of exons 7 and 8 of *VCAN*.

In the present study, the lead SNPs in chr5q14 were inversely associated with small vessel stroke, and, therefore, our study does not provide support for any vascular-mediated mechanism underlying the association of *VCAN* with FA and MD.

The consistent correlation between MD, FA, WMHV, and MRI-confirmed lacunar stroke further contributes to the evidence that there is a shared polygenic component in the disease mechanisms of small vessel disease and alterations of the white matter.

In addition, there was an association between MD and major depressive disorder in the polygenic risk score analysis. This finding is in line with previous imaging studies that found a direct association between reduced structural integrity and major depressive disorder.^32^ The correlation between MD and major depressive disorder did not reach genome-wide significance in the LD score regression, although the effect was in the same direction. A possible explanation for this discrepancy is that even though the current study is the largest study published on genetics of white matter microstructural integrity to date, the sample size is still relatively small for GWAS. The cases contributing to the major depressive disorder had a broad range of ages of disease onset. At this point, it is not possible to determine whether the shared genetic component with MD is because of age-related changes or otherwise. This will be an important point of further enquiry.

In the current analysis, Alzheimer disease was significantly associated with WMHV in the polygenic risk score analysis but not with the white matter microstructural integrity measures. Furthermore, the top SNPs for WMHV were not statistically significant associated with FA and MD, although the effect estimates were in the expected directions. An explanation for the discrepancies might be that there are differences in the information FA, MD, and WMHV capture. FA and MD in the present study represent microstructural integrity within both normal-appearing white matter, and WMH and microstructural integrity is lower in the latter.^33^ Decreased microstructural integrity is known to precede WMH.^34^ Therefore, the WMH assessed in this relatively healthy population may reflect more severe disease than the microstructural integrity. On the other hand, pathological studies have shown that WMHs are heterogenic, that is, they represent different degrees of tissue damage, but this is not captured by the WMHV measure.^35^ Another explanation for the discrepancies might be the lack of statistical power because of a combination of only mild detectable disease in this relatively young and healthy population and a sample size that is still small for a GWAS.

Strengths of the present study include the large sample size with high-quality standardized research MRI scans.

This present study is limited to white participants of European genetic descent because only a small fraction (2%–3%) of the participants were of non-European descent. Thus, the results may not be applicable to other populations. Another limitation is that we were not able to replicate our findings in independent samples because unfortunately no large-scale replication resources are currently available.

We identified a novel locus that is genome-wide associated with microstructural integrity of the white matter in the brain measured as FA and MD. The results contribute to the growing evidence that mechanisms underlying white matter alterations are shared with cerebrovascular disease and highlight that inherited differences in white matter microstructure, possibly age related, impact on multiple diseases.

## Acknowledgments

We thank the International Genomics of Alzheimer Project (IGAP), the Psychiatric Genomics Consortium, the genetics of magnetic resonance imaging–confirmed lacunar stroke collaboration, and the Intracerebral Hemorrhage Genetics collaboration for providing summary results data for these analyses. The investigators within IGAP contributed to the design and implementation of IGAP and provided data but did not participate in the analysis or writing of this report. IGAP was made possible by the generous participation of the control subjects, the patients, and their families.

## Sources of Funding

This project has received funding from the European Union Horizon 2020 research and innovation program under grant agreement number 667375. This work was, in part, supported by a British Heart Foundation Programme Grant (RG/16/4/32218). Dr Rutten-Jacobs was supported by a British Heart Foundation Immediate Research Fellowship (FS/15/61/31626). Dr Tozer is supported by the Cambridge Universities National Institute for Health Research (NIHR) Comprehensive Biomedical Research Centre. Dr Dichgans received funding from the European Union Horizon 2020 research and innovation program under grant agreement number 666881 (SVDs@target) and from the German Research Foundation through the Collaborative Research Centres 1123 (B3) and the Munich Cluster for Systems Neurology (EXC 1010 SyNergy). H.S. Markus is supported by an NIHR Senior Investigator award, and his work is supported by the Cambridge Universities NIHR Comprehensive Biomedical Research Centre. International Genomics of Alzheimer Project: the i–Select chips was funded by the French National Foundation on Alzheimer disease and related disorders. European Alzheimer Disease Initiative was supported by the LABEX (laboratory of excellence program investment for the future) Development of Innovative Strategies for a Transdisciplinary Approach to Alzheimer's Disease (DISTALZ) grant, Inserm, Institut Pasteur de Lille, Université de Lille 2, and the Lille University Hospital. Genetic and Environmental Risk in Alzheimer's Disease (GERAD) was supported by the Medical Research Council (grant No. 503480), Alzheimer Research UK (grant No. 503176), the Wellcome Trust (grant No. 082604/2/07/Z), the and German Federal Ministry of Education and Research: Competence Network Dementia grant numbers 01GI0102, 01GI0711, and 01GI0420. Cohorts for Heart and Aging Research in Genomic Epidemiology Consortium (CHARGE) was partly supported by the National Institutes of Health (NIH)/National Institute on Aging grant R01 AG033193 and the National Institute on Aging AG081220 and Age, Gene/Environment Susceptibility Study (AGES) contract N01–AG–12100, the National Heart, Lung, and Blood Institute grant R01 HL105756, the Icelandic Heart Association, and the Erasmus Medical Center and Erasmus University. Alzheimer's Disease Genetics Consortium was supported by the NIH/National Institute on Aging grants U01 AG032984, U24 AG021886, and U01 AG016976 and the Alzheimer Association grant ADGC–10 to 196728. Genetics of magnetic resonance imaging (MRI)–confirmed lacunar stroke collaboration: the genetics of MRI-confirmed lacunar stroke project contains samples derived from the National Institute of Neurological Disorders and Stroke Stroke Genetics Network (SIGN-NINDS) study, the Wellcome Trust Case Control Consortium 2 stroke study, and DNA Lacunar (UK Young Lacunar Stroke DNA Study). The Stroke Genetics Network (SiGN) study was funded by a cooperative agreement grant from the US National Institute of Neurological Disorders and Stroke, NIH (U01 NS069208). Collection of the DNA Lacunar was primarily supported by the Wellcome Trust (WT072952) with additional support from the Stroke Association (TSA 2010/01). Genotyping of the DNA Lacunar samples was supported by a Stroke Association Grant (TSA 2013/01). The principal funding for the WTCCC2 stroke study was provided by the Wellcome Trust, as part of the Wellcome Trust Case Control Consortium 2 project (085475/B/08/Z, 085475/Z/08/Z, and WT084724MA).

## Disclosures

H.S. Markus received personal compensation for lectures from AstraZeneca. The other authors report no disclosures.

## Supplementary Material
