# Supplementary material for: Genetic Study of White Matter Integrity in UK Biobank (N=8448) and the Overlap With Stroke, Depression, and Dementia
Source: Stroke. 2018 May 11;49(6):1340–7. doi: 10.1161/STROKEAHA.118.020811 (PMC5976227; doi:10.1161/STROKEAHA.118.020811)
Supplement: Supplementary file 1 [file str-49-1340-s001.pdf]

SUPPLEMENTAL MATERIAL

Genetic study of white matter integrity in UK Biobank (N=8,448) and the overlap with stroke, depression and dementia.

Loes C.A. Rutten-Jacobs, PhD[1,3]; Daniel J. Tozer, PhD[1]; Marco Duering, MD[2]; Rainer Malik, PhD[2]; Martin Dichgans, MD[2]; Hugh S. Markus, DM[1]; Matthew Traylor, PhD[1]

[1] Department of Clinical Neurosciences, Stroke Research Group, University of Cambridge, UK
[2] Institute for Stroke and Dementia Research, Klinikum der Universität München, Germany
[3] German Center for Neurodegenerative diseases (DZNE), Population Health Sciences, Bonn, Germany

Corresponding authors:

Dr Loes Rutten-Jacobs, German Center for Neurodegenerative diseases (DZNE), Population Health Sciences, Sigmund-Freud-Straße 27, 53127 Bonn, Germany. Email: Loes.Rutten-Jacobs@dzne.de; Telephone: +49 (0)22843302954

Dr Matthew Traylor, Department of Clinical Neurosciences, Stroke Research Group, University of Cambridge, Cambridge Biomedical Campus, R3, Cambridge, CB2 0QQ, UK. Email: MT628@medschl.cam.ac.uk; Telephone: +44 (0)1223256608

Contents

Figure I Flow chart study population
Figure II Quantile-Quantile (QQ) plots for FA (A), MD (B) and WMH (C).
Figure III Regional plot for genome-wide significant signals for FA
Figure IV Regional plot for genome-wide significant signals for MD
Figure V Regional plots for independent genome-wide significant signals for WMH
Figure VI Regional plots of the chr5q14 locus for FA showing univariable association results (A) and association results conditioned on the top SNP (rs67827860).
Figure VII Regional plots of the chr5q14 locus for MD showing univariable association results (A) and association results conditioned on the top SNP (rs13164785).
Table I History of neurodegenerative disease self-report and medical record codes in UK Biobank
Table II Overview of data used for the analysis of secondary traits
Table III Studies included in the MRI-confirmed lacunar stroke collaboration
Table IV Joint modelling of Independent Signals at chr5q14 locus
Table V Association of lead SNPs for WMH with FA and MD
Supplemental Methods Alzheimer's disease GWAS data
Supplemental references

Figure I Flow chart study population

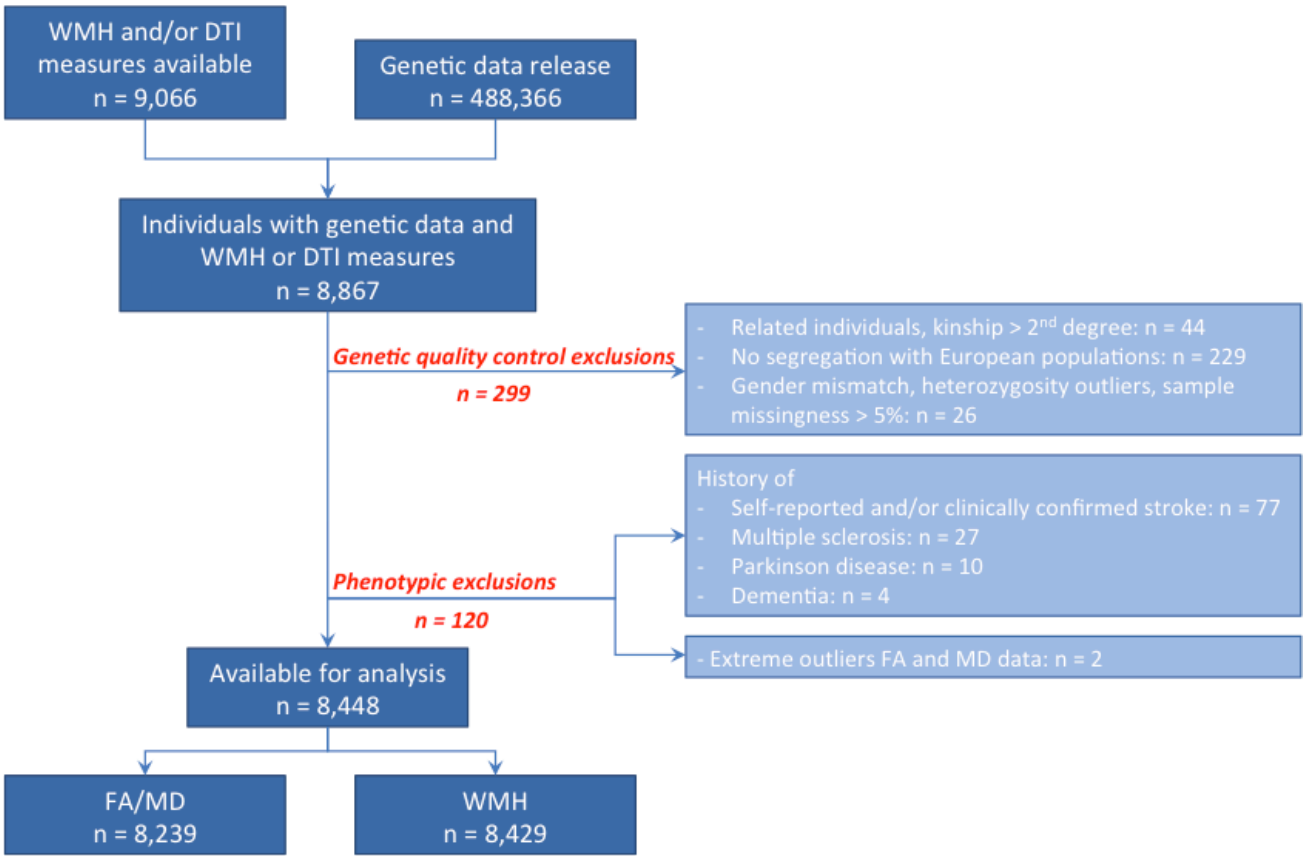

Figure II Quantile-Quantile (QQ) plots for FA (A), MD (B) and WMH (C).

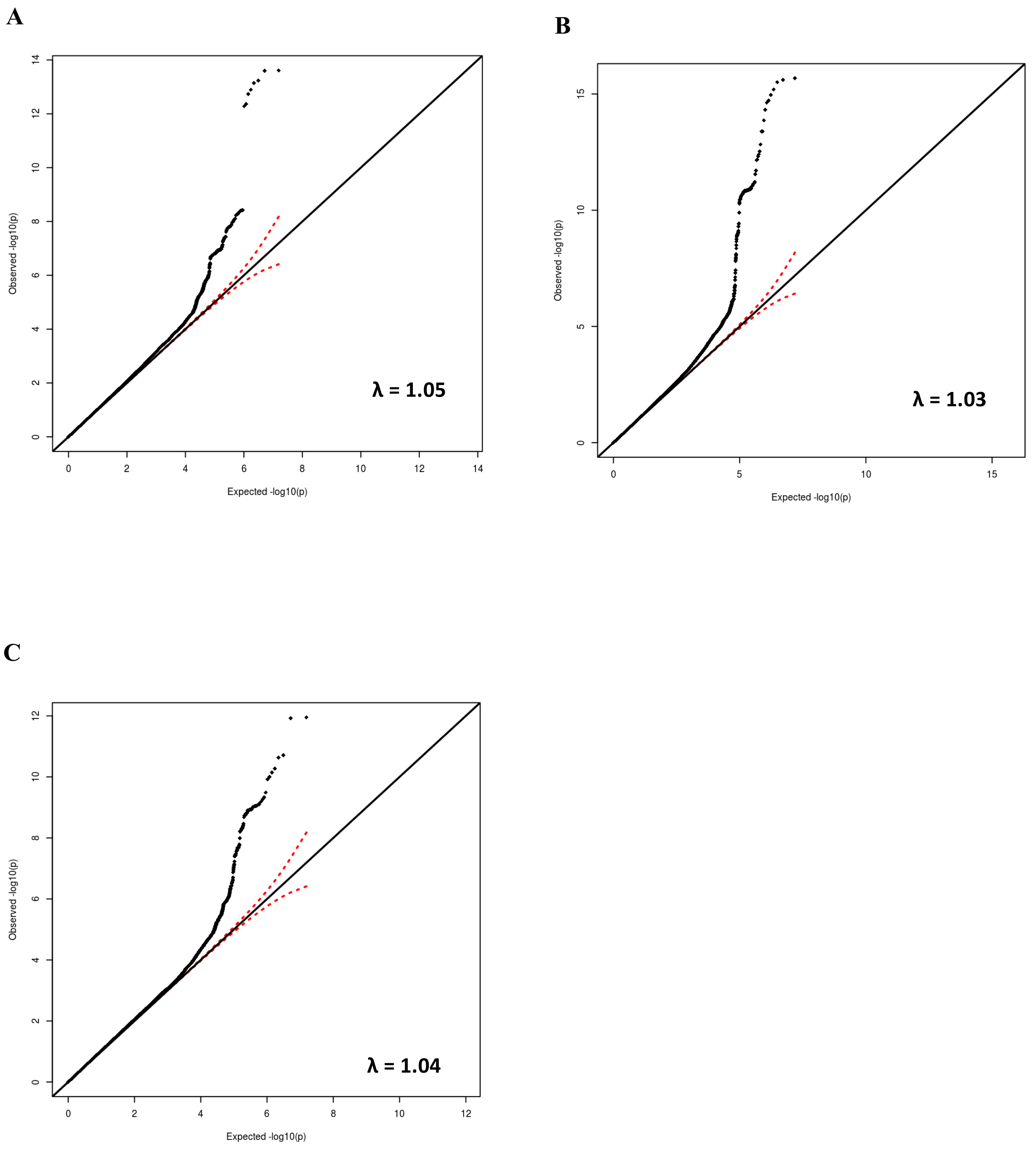

Shown in black are the observed versus expected –log(p-value) distributions. The red line shows the expected (null) distribution of the statistic.

Figure III Regional plot for genome-wide significant signals for FA

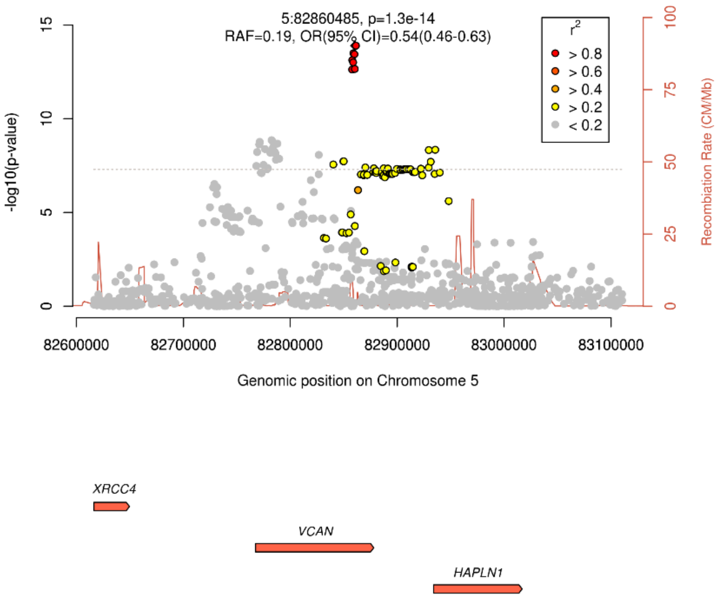

Shown is the region for the top signal ± 250kb. The colour scale corresponds to the r2 value for the SNP and the top SNP.

Figure IV Regional plot for genome-wide significant signals for MD

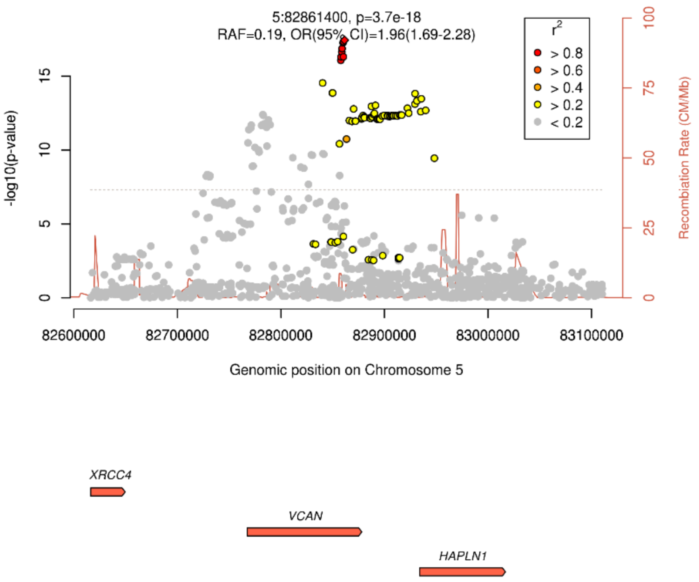

Shown is the region for the top signal ± 250kb. The colour scale corresponds to the r^2 value for the SNP and the top SNP.

Figure V Regional plots for independent genome-wide significant signals for WMH

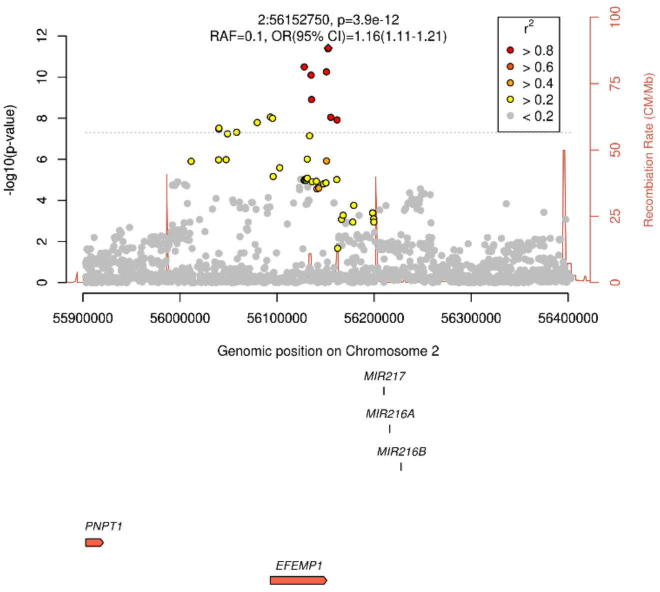

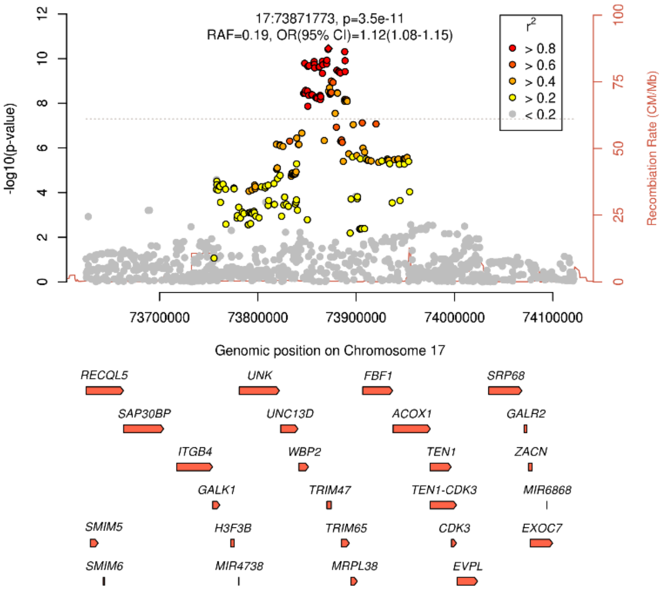

Shown is the region for the top signal ± 250kb. The colour scale corresponds to the r^2 value for the SNP and the top SNP.

Figure VI Regional plots of the chr5q14 locus for FA showing univariable association results (A) and association results conditioned on the top SNP (rs67827860).

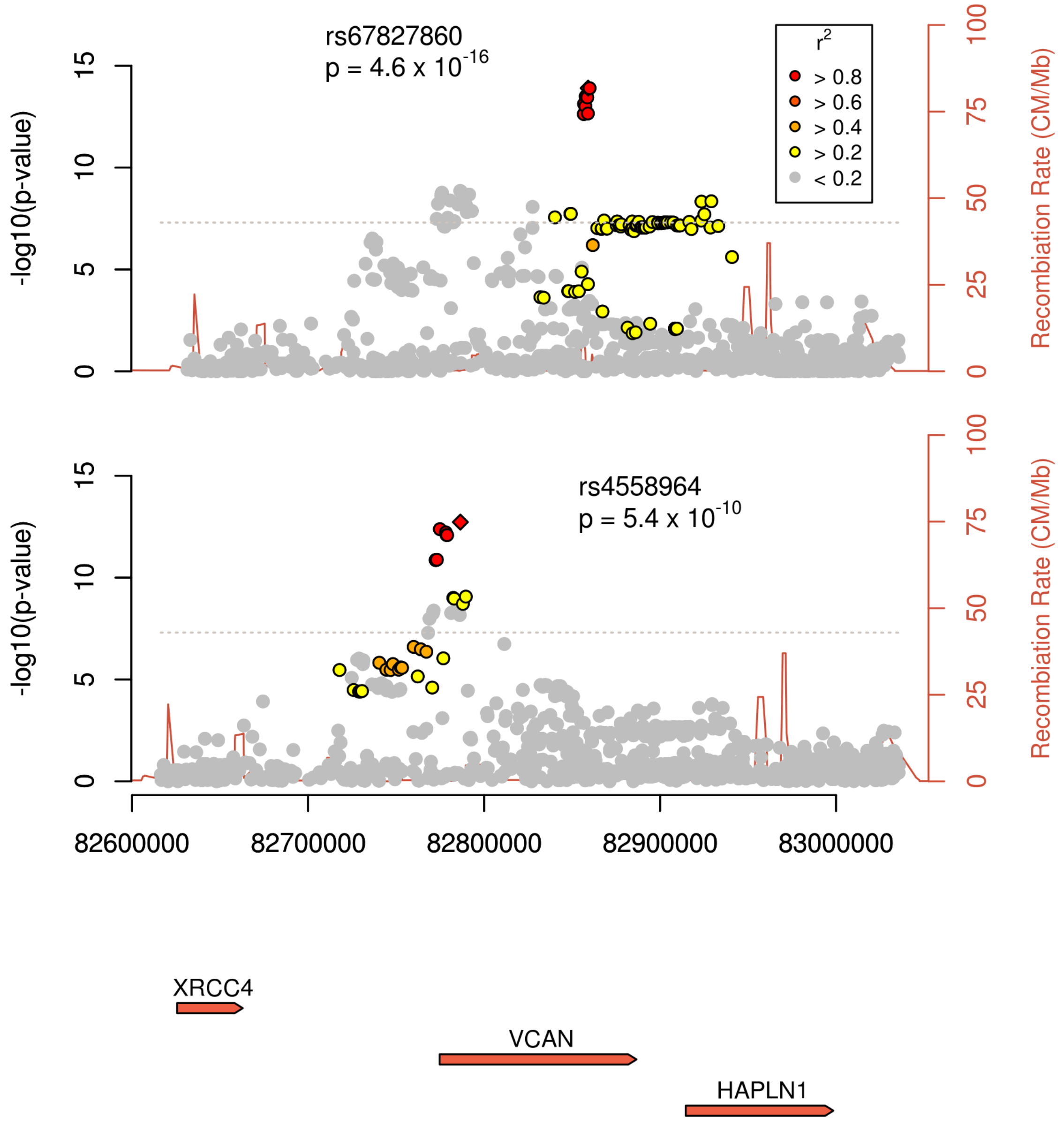

Shown is the region for the top signal ± 250kb. The colour scale corresponds to the r^2 value for the SNP and the top SNP.

Figure VII Regional plots of the chr5q14 locus for MD showing univariable association results (A) and association results conditioned on the top SNP (rs13164785).

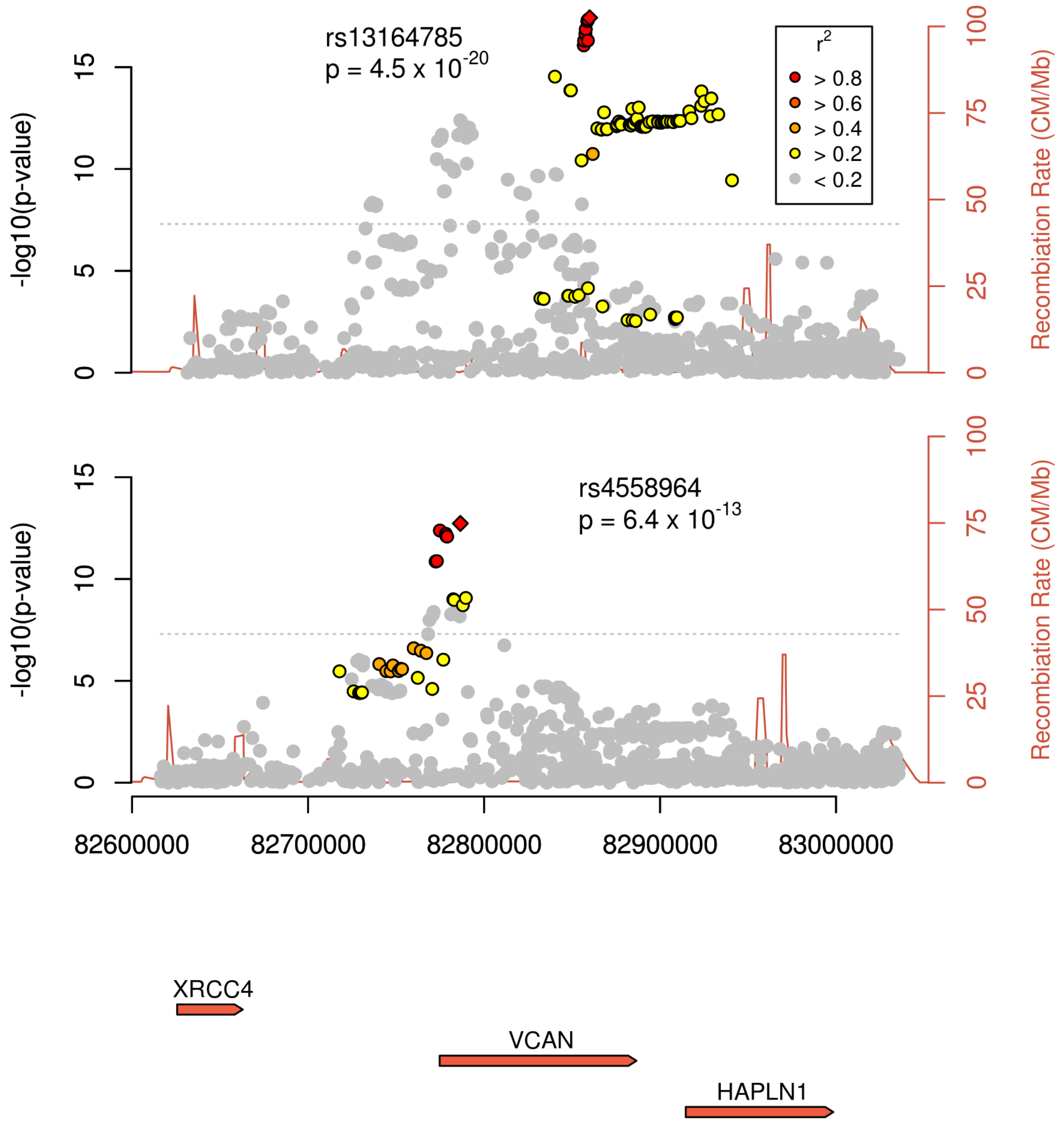

Shown is the region for the top signal ± 250kb. The colour scale corresponds to the r^2 value for the SNP and the top SNP.

Table I History of neurodegenerative disease self-report and medical record codes in UK Biobank

	**Self-report UK Biobank code**	**ICD-9**	**ICD-10**
Stroke	1081, 1086, 1491, 1583	430, 431, 434, 436	I60, I61, I63, I64
Multiple Sclerosis	1261	340	G35
Parkinson's disease	1262	332	G20
Dementia	1263	290	F00-F03
Any other neurodegenerative disease	1397	341	G30-G32, G36, G37

Table II Overview of data used for the analysis of secondary traits

Study	Trait	Reference	Sample size cases/controls	Data publicly available
International Genomic's of Alzheimer's Project (IGAP)	Alzheimer's Disease	[1]	17008/37154	Yes
Psychiatric Genomics Consortium (PGC)	Major depressive disorder	[2]	9240/9519	Yes
Genetics of MRI-confirmed lacunar stroke collaboration*	MRI-confirmed lacunar stroke	[3]	2191/27297	No
Intracerebral Hemorrhage Genetics Collaboration†	Intracerebral haemorrhage	[4]	1545/1481	Yes

Overview of studies with available summary statistics from related traits used for the look-ups, LD score regression and polygenic risk score analysis.

*The Genetics of MRI-confirmed lacunar stroke collaboration is an ongoing international collaboration and is still expanding in size. The published study on MRI-confirmed lacunar stroke has been performed on a previous version of the dataset containing a smaller number of cases.

†Intracerebral Haemorrhage data were downloaded from the Cerebrovascular Disease Knowledge Portal (http://cerebrovascularportal.org/informational/downloads)

Table III Studies included in the MRI-confirmed lacunar stroke collaboration

Cohorts	Cases	Controls
DNA LACUNAR	917	
GENESIS (SR/ADDS)	299	
PRESERVE	46	
UK-WTCCC2	250	
GERMANY-WTCCC2	37	
MILANO	9	
ASGC	23	
SIGN:BRAINS	5	
SIGN:GEOS	4	
SIGN:GCNKSS	27	
SIGN:MIAMISR	13	
SIGN:GASROS	27	
SIGN:ISGS	28	
SIGN:KRAKOW	7	
SIGN:LEUVEN	45	
SIGN:BASICMAR	36	
SIGN:SAHLSIS	31	
SIGN:SPS3_EUR	345	
SIGN:GRAZ	42	
1958 BIRTH COHORT, NATIONAL BLOOD SERVICE		5175
DNA LACUNAR		968
KORA		797
SIGN:HRS		9286
SIGN:GRAZ		816
SIGN:LEUVEN		453
SIGN:KRAKOW		716
SIGN:ADHD		411
SIGN:MALMO		1362
SIGN:OAI		3201
SIGN:GEOS		519
SIGN:ASGS		1200
SIGN:HABC		1586
SIGN:INMA		807
**TOTAL EUROPEAN**	**2191**	**27297**

Details of the individual studies has been previously described.[3, 5]
Standard thorough quality control was performed on each dataset separately. The data were then aligned to the forward strand and imputed to the HRC reference panel. SNPs with MAF<0.01 or INFO<0.5 were then removed and association analysis was carried out using RVTESTS[6] including age, sex, study group, and the first 10 principal components. The inflation of test statistics (λ) was equal to the inflation expected for the sample size.

Table IV Joint modelling of Independent Signals at chr5q14 locus

Trait	rsID	Chr	Position (bp)	Effect allele	Non-effect allele	INFO	EAF	Beta	SE	P-value
FA	rs67827860	5	82860485	T	C	0.997	0.19	-0.644	0.079	4.59×10^{-16}
	rs4558964	5	82786549	G	A	0.993	0.26	0.439	0.071	5.41×10^{-10}
MD	rs13164785	5	82861400	G	T	0.995	0.19	0.711	0.077	4.54×10^{-20}
	rs4558964	5	82786549	G	A	0.993	0.26	-0.497	0.069	6.39×10^{-13}

Abbreviations: FA, fractional anisotropy; MD, mean diffusivity; Chr, chromosome; INFO, imputation quality score; EAF, effect allele frequency.

Position is reported on GRCh37.

Table V Association of lead SNPs for WMH with FA and MD

Trait	rsID	Chr	Position (bp)	Effect allele	Non-effect allele	Beta	SE	P-value
FA	rs146896516	2	56152750	A	C	-0.159	0.102	0.12
	rs3744020	17	73871773	A	G	-0.027	0.079	0.73
MD	rs146896516	2	56152750	A	C	0.292	0.100	0.003
	rs3744020	17	73871773	A	G	0.097	0.078	0.21

Abbreviations: FA, fractional anisotropy; MD, mean diffusivity; Chr, chromosome; EAF, effect allele frequency.

Position is reported on GRCh37.

Supplemental Methods

Alzheimer's disease GWAS data

International Genomics of Alzheimer's Project (IGAP) is a large two-stage study based upon genome-wide association studies (GWAS) on individuals of European ancestry. In stage 1, IGAP used genotyped and imputed data on 7,055,881 single nucleotide polymorphisms (SNPs) to meta-analyse four previously-published GWAS datasets consisting of 17,008 Alzheimer's disease cases and 37,154 controls (The European Alzheimer's disease Initiative – EADI; the Alzheimer Disease Genetics Consortium – ADGC; The Cohorts for Heart and Aging Research in Genomic Epidemiology consortium – CHARGE; The Genetic and Environmental Risk in AD consortium – GERAD). In stage 2, 11,632 SNPs were genotyped and tested for association in an independent set of 8,572 Alzheimer's disease cases and 11,312 controls. Finally, a meta-analysis was performed combining results from stages 1 & 2.

Supplemental References

1. Lambert JC, Ibrahim-Verbaas CA, Harold D, Naj AC, Sims R, Bellenguez C, et al. Meta-analysis of 74,046 individuals identifies 11 new susceptibility loci for alzheimer's disease. *Nat Genet*. 2013;45:1452-1458
2. Major Depressive Disorder Working Group of the Psychiatric GC, Ripke S, Wray NR, Lewis CM, Hamilton SP, Weissman MM, et al. A mega-analysis of genome-wide association studies for major depressive disorder. *Mol Psychiatry*. 2013;18:497-511
3. Traylor M, Rutten-Jacobs LC, Thijs V, Holliday EG, Levi C, Bevan S, et al. Genetic associations with white matter hyperintensities confer risk of lacunar stroke. *Stroke*. 2016;47:1174-1179
4. Woo D, Falcone GJ, Devan WJ, Brown WM, Biffi A, Howard TD, et al. Meta-analysis of genome-wide association studies identifies 1q22 as a susceptibility locus for intracerebral hemorrhage. *Am J Hum Genet*. 2014;94:511-521
5. Meschia JF, Arnett DK, Ay H, Brown RD, Jr., Benavente OR, Cole JW, et al. Stroke genetics network (sign) study: Design and rationale for a genome-wide association study of ischemic stroke subtypes. *Stroke*. 2013;44:2694-2702
6. Zhan X, Hu Y, Li B, Abecasis GR, Liu DJ. Rvtests: An efficient and comprehensive tool for rare variant association analysis using sequence data. *Bioinformatics*. 2016;32:1423-1426
